# Alternative Signaling Pathways as Potential Therapeutic Targets for Overcoming EGFR and c-Met Inhibitor Resistance in Non-Small Cell Lung Cancer

**DOI:** 10.1371/journal.pone.0078398

**Published:** 2013-11-04

**Authors:** Jason T. Fong, Ryan J. Jacobs, David N. Moravec, Srijayaprakash B. Uppada, Gregory M. Botting, Marie Nlend, Neelu Puri

**Affiliations:** 1 Department of Biomedical Sciences, University of Illinois College of Medicine, Rockford, Illinois, United States; 2 Thermo Fisher Scientific, Rockford, Illinois, United States of America; University of Alabama at Birmingham, United States of America

## Abstract

The use of tyrosine kinase inhibitors (TKIs) against EGFR/c-Met in non-small cell lung cancer (NSCLC) has been shown to be effective in increasing patient progression free survival (PFS), but their efficacy is limited due to the development of resistance and tumor recurrence. Therefore, understanding the molecular mechanisms underlying development of drug resistance in NSCLC is necessary for developing novel and effective therapeutic approaches to improve patient outcome. This study aims to understand the mechanism of EGFR/c-Met tyrosine kinase inhibitor (TKI) resistance in NSCLC. H2170 and H358 cell lines were made resistant to SU11274, a c-Met inhibitor, and erlotinib, an EGFR inhibitor, through step-wise increases in TKI exposure. The IC_50_ concentrations of resistant lines exhibited a 4–5 and 11–22-fold increase for SU11274 and erlotinib, respectively, when compared to parental lines. Furthermore, mTOR and Wnt signaling was studied in both cell lines to determine their roles in mediating TKI resistance. We observed a 2–4-fold upregulation of mTOR signaling proteins and a 2- to 8-fold upregulation of Wnt signaling proteins in H2170 erlotinib and SU11274 resistant cells. H2170 and H358 cells were further treated with the mTOR inhibitor everolimus and the Wnt inhibitor XAV939. H358 resistant cells were inhibited by 95% by a triple combination of everolimus, erlotinib and SU11274 in comparison to 34% by a double combination of these drugs. Parental H2170 cells displayed no sensitivity to XAV939, while resistant cells were significantly inhibited (39%) by XAV939 as a single agent, as well as in combination with SU11274 and erlotinib. Similar results were obtained with H358 resistant cells. This study suggests a novel molecular mechanism of drug resistance in lung cancer.

## Introduction

EGFR and c-Met are both highly expressed in NSCLC tumors and share common signaling pathways [1]–[3]. While TKIs against EGFR and c-Met are on the cutting-edge of cancer therapy, their individual efficacies are limited [4] due to the development of resistance [5]. c-Met amplification accounts for more than 20% of acquired resistance to EGFR TKIs in NSCLC both *in vitro* and *in vivo* [6], [7]. Furthermore, development of secondary ”gatekeeper” mutation T790M accounts for 50% of all acquired resistance to EGFR TKIs both *in vitro* and *in vivo* [8]. Also, its presence prior to treatment with TKIs results in primary resistance to EGFR TKI therapy [9]. Therefore, to explore mechanisms of resistance it is important to conduct additional *in vitro* studies for determining target proteins responsible for TKI resistance in NSCLC.

SU11274 is an ATP-competitive small molecule inhibitor of the catalytic activity of c-Met [10] and is effective against NSCLC [11]. Tivantinib, a c-Met TKI which inhibits tumor growth in mice [12], is currently in Phase III clinical trials and has been shown to increase PFS from 9.7 to 16.1 weeks when given in combination with erlotinib [13], [14]. In these trials, only certain patient subsets (KRAS mutants, non-squamous histology and EGFR wild-type status) exhibited significantly increased PFS [14], suggesting that new TKIs need to be added to this combination. Additionally, treatment with a combination of MetMab (anti c-Met mAb) and erlotinib reduced the risk of death by 3-fold in only a subset of patients positive for c-Met expression [15]. While the use of combined therapy modalities may limit the ability of tumors to develop resistance [7], understanding the mechanism of resistance is the best approach for improving targeted therapy [16].

Studies by our group and others indicate that c-Met and EGFR have considerable crosstalk which increases efficacy for TKI combinations *in vitro* [1], [17]. This is due to the fact that HGF can transactivate EGFR and phosphorylation of EGFR can activate c-Met resulting in synergistic effects on tumor growth [18]–[20]. Therefore, we investigated a novel therapeutic approach for overcoming resistance to EGFR, c-Met and EGFR/c-Met TKI combination therapies in NSCLC. To further understand how cells develop this resistance we developed H2170 and H358 NSCLC cell lines with acquired resistance to TKIs of c-Met, EGFR and a combination of both. These cell lines were chosen because they express high levels of EGFR and c-Met, are synergistically inhibited by EGFR/c-Met TKIs and do not have pre-existing EGFR or c-Met resistance causing mutations.

Previous studies have demonstrated increased efficacy with combination therapies when compared to monotherapies [13], [14], [21]–[24]. The mTOR inhibitor rapamycin is able to cooperate with c-Met inhibitor PHA665752 in NSCLC [25]. Further, using erlotinib and rapamycin or everolimus (an orally administered derivative of rapamycin) in combination has shown synergistic effects on cell viability, proliferation and autophagy [26], [27]. This combination was also shown to restore gefitinib sensitivity [28]. However, these studies only administered EGFR and mTOR inhibitors in combination. In our studies, we have found that mTOR inhibitors can increase the efficacy of EGFR/c-Met TKI combination therapy for NSCLC *in vitro*. Furthermore, it has been suggested that crosstalk between EGFR and the Wnt pathway may participate in the onset and progression of tumorigenesis and crosstalk between ligands of separate RTK mediated pathways may facilitate resistance to TKIs [29]. Hyperactivity of Wnt in breast cancer has been shown to transactivate EGFR and conversely, activated EGFR may contribute to increased effect of the canonical Wnt pathway [30]–[33]. Additionally, studies on patient tumor sections have shown a positive correlation between EGFR activating mutations and nuclear accumulation of β-catenin in primary NSCLC [34]. It has also been shown that the Wnt/β-catenin pathway has a substantial role in cell maintenance, pathogenesis and resistance following EGFR inhibition in NSCLC [35].

Our data demonstrates that the addition of Wnt inhibitors to TKIs SU11274 and erlotinib results in significantly decreased viability in cell lines with acquired resistance to combination EGFR/c-Met TKI therapy. We suggest that activation of alternative signaling pathways is a possible molecular mechanism of drug resistance in NSCLC, utilizing c-Met, EGFR, mTOR and Wnt inhibitors could greatly improve lung cancer patient prognosis and be the basis for new clinical trials.

## Materials and Methods

### Reagents and Antibodies

SU11274: [(3Z)-N-(3-chlorophenyl)-3-({3,5-dimethyl-4-[(4-methylpiperazin-1-yl) carbonyl]1H-pyrrol-2-yl}methylene)-N-methyl-2-oxo-2,3-dihydro-1H-indole-5-sulfonamide] and XAV939: [3,5,7,8-tetrahydro-2-[4-(trifluoromethyl)phenyl]-4H-thiopyrano[4,3-d]pyrimidin-4-one] were obtained from Sigma-Aldrich (St. Louis, MO). Erlotinib and Everolimus were obtained from LC Laboratories (Woburn, MA). Tivantinib was obtained from Chemietek (Indianapolis, IN). All inhibitors were suspended in DMSO and kept in 0.1ml aliquots at –20°C. HGF was obtained from Peprotech (Rocky Hill, NJ) and EGF was obtained from Cell Signaling Technology (Beverly, MA). Phosphospecific rabbit mAbs for p-EGFR (Tyr1068, Clone D7A5), p-mTOR (Ser2448, Clone D9C2) and p-4E-BP1 (Thr37/46, Clone 2855) were obtained from Cell Signaling Technology, Inc (Beverly, MA). Phosphospecific rabbit polyclonal antibodies for p-ERK1/2 (Thr202/Tyr204, 2532) and p-p70S6K (Tyr421/Ser424, 9208) were obtained from Cell Signaling Technology. A rabbit polyclonal antibody for p-c-Met (Tyr 1003, 44882G) was obtained from Invitrogen (Carlsbad, CA). A mouse mAb for active β-catenin (clone 8E7, 05-665) was obtained from Millipore (Billerica, MA). For Wnt signaling studies, all antibodies were obtained from Wnt signaling antibody sampler kit from Cell Signaling Technology (2915). Unphosphorylated rabbit mAbs for p70S6K (2708) and mTOR (2983) were obtained from Cell Signaling Technology. Unphosphorylated c-Met (C-12, sc-10) and EGFR (1005, sc-03) were obtained from Santa Cruz Biotechnology (Santa Cruz, CA). A mouse mAb for β-actin (A5441) was obtained from Sigma-Aldrich (St. Louis, MO). Mouse (7076) and rabbit (7074) IgG secondary antibodies were obtained from Cell Signaling Technology. All antibodies were used as described by the manufacturer's instructions.

### Cell Lines and Cell Culture

H358 and H2170 NSCLC cell lines were obtained from the American Type Culture Collection (Rockville, MD, CRL-5807 and CRL-5928, respectively) and cultured according to their instructions. Cell lines were incubated at 37°C and 7% CO_2_ and maintained in Roswell Park Memorial Institute (RPMI) medium (Thermo Fisher Scientific, Pittsburg, PA, Cat No: SH3002701) supplemented with 10% (v/v) fetal bovine serum (Atlanta Biologicals, Lawrenceville, GA) and 1% (v/v) antibiotic/antimycotic (Invitrogen, Cat No: 15240). For studying the effects of erlotinib and SU11274 as single therapeutic agents, as well as in combination, H358 and H2170 cells were plated in 6-well plates and treated after 24 hours. Then, MTT viability assays and western blots were performed as described below. IC_50_ values for each cell line were calculated using Sigma Plot V12.0 (SYSTAT Software, San Jose, CA).

### MTT Cell Viability Assays

Cell viability was measured by an MTT colorimetric dye reduction assay (Sigma, St. Louis, MO) using 3-(4,5-dimethylthiazol-2-yl)-2,5-diphenyltetrazolium bromide) according to the manufacturer's instructions. Each experiment was performed in 96-well plates in replicates of six for each treatment condition and repeated three times. Cells were plated at 5000 cells/well and treated with inhibitors after 24 hours of growth. At 96 hours after plating, percent cell viability was calculated relative to control by measuring absorbance at a wavelength of 570 nm. Cells treated with tivantinib were exposed only for 24 hours, after which drug was removed. Cells were then washed and incubated for an additional 72 hours as described by earlier investigators [12].

### Preparation of Cell Lysates and Western Blotting

Cells were grown and lysed as described previously [36], [37] in buffer containing 20 mM Tris (pH, 8.0), 150 mM NaCl, 10% glycerol, 1% NP-40, 0.42% NaF, 1 mM phenylmethylsulfonyl fluoride, 1 mM sodium orthovanadate, and 10 mM protease inhibitor cocktail (Sigma- Aldrich). Cell lysates were separated by 7.5% or 10% SDS-PAGE under reducing conditions. Proteins were then transferred to immobilization membranes (Bio-Rad Laboratories, Hercules, CA). The membranes were then probed with the aforementioned antibodies. Blots were visualized using an enhanced chemiluminescence kit (Thermo Fisher Scientific, Rockford, IL) and quantification of modulation of different proteins was performed using NIH ImageJ software.

### Specific phosphorylation of c-Met/EGFR and other signaling pathways via HGF/EGF and their inhibition

Cells were deprived of growth factors by incubation for 24 hours in serum-free medium containing 0.5% BSA with or without inhibitors. Following treatment, cells were stimulated with 40 ng/mL HGF for 7.5 minutes or 5 ng/mL EGF for 5 minutes at 37°C. After preparing lysates, western blotting was performed as described above.

### Immunofluorescence

10,000 cells were plated on glass chamber slides (Lab-TeK, Scotts Valley, CA) and allowed to adhere for 24 hours in serum-free medium with 0.5% BSA. Cells were then treated with EGF for 15 minutes, fixed with 1∶1 acetone:methanol and visualized with p-EGFR (Y1068) primary antibody, anti-rabbit DyLight 488 (green) secondary antibody (Thermo Fisher Scientific) and Hoechst dye for nuclear staining (blue) using a Zeiss Axio Observer Z1 fluorescent microscope. NIH ImageJ software was used to measure total cell fluorescence intensity over 8 microscopic fields per condition and values were averaged.

### DNA Sequencing

5 million cells were plated on 150 mm diameter dishes and allowed to adhere and grow in media as described above. DNA was extracted using the Qiagen DNeasy® Blood & Tissue kit (cat. no. 69504) following manufacturer's instructions. PCR was then performed to amplify exons 18–21 of EGFR using primers described by Paez et al [38], using the AmpliTaq Gold® PCR Master Mix (Applied Biosystems, cat. no. 4327058). The PCR products were purified using the GeneJET™ PCR Purification Kit (cat. no. K0701) and were then sequenced at the University of Illinois DNA Services Facility.

### Statistical analysis

Statistical analyses were carried out using SPSS 17.0 software. Repeated measures of ANOVA with multiple pairwise comparisons and custom contrasts with Bonferroni adjustments were performed. Statistical significance was determined with α at 0.05. To confirm the differences between treatments a paired two-tailed Student's *t*-test was also used. For all analyses, a p-value of less than 0.05 was considered to be statistically significant.

## Results

### Establishment of drug resistant cell lines

To identify appropriate concentrations of SU11274, erlotinib and a combination of both TKIs for the development of resistant cell lines, H2170 and H358 cell lines were treated with progressively increasing concentrations of SU11274 (2.5–17 µM) [1], erlotinib (0. 5–14 µM) [39], or both SU11274 (1.25–13.5 µM) and erlotinib (0.25–9 µM) for 96 hours. H2170 and H358 cell lines were selected because they do not have EGFR TK or c-Met mutations. IC_50_ values for individual TKIs or a combination were determined for each cell line (**Table 1**). Cells were then treated with increasing concentrations of SU11274 [1], erlotinib [39] or a combination for several weeks after which five individual resistant clones were isolated from single cells, expanded and then checked for stable resistance after each serial passage (once per week) [40]. Resistant cells were grown in the absence of TKIs for 12 passages (12 weeks) and were found to retain resistance. Resistant clones from cell lines described in **Table 1**, with IC_50_ concentrations (determined as described in materials and methods section) 4–5-fold higher for SU11274, 11–22-fold higher for erlotinib, and 6–8-fold higher for SU11274 and 15–30-fold higher for erlotinib in combination, were isolated and selected for further studies. Lower concentrations were necessary for combination resistance, since we observed enhanced effects of erlotinib and SU11274 when they were used in combination. Similar results were obtained with other resistant clones (data not shown).

**Table 1:** IC_50_ of RTKIs and Combinations for Parental and Resistant NSCLC cell lines.

Cell line	SU11274	Erlotinib	Combination
H2170 Parental	2.5 µM	0.5 µM	1.25 µM SU11274/0.25 µM Erlotinib
H2170 Resistant	12 µM	11 µM	7.5 µM SU11274/7.5 µM Erlotinib
H358 Parental	2.5 µM	1 µM	1.25 µM SU11274/0.5 µM Erlotinib
H358 Resistant	11 µM	11 µM	10 µM SU11274/7.5 µM Erlotinib

### SU11274 resistant (SR) cells exhibit decreased sensitivity to the oral c-Met inhibitor, tivantinib

**Figure 1:**
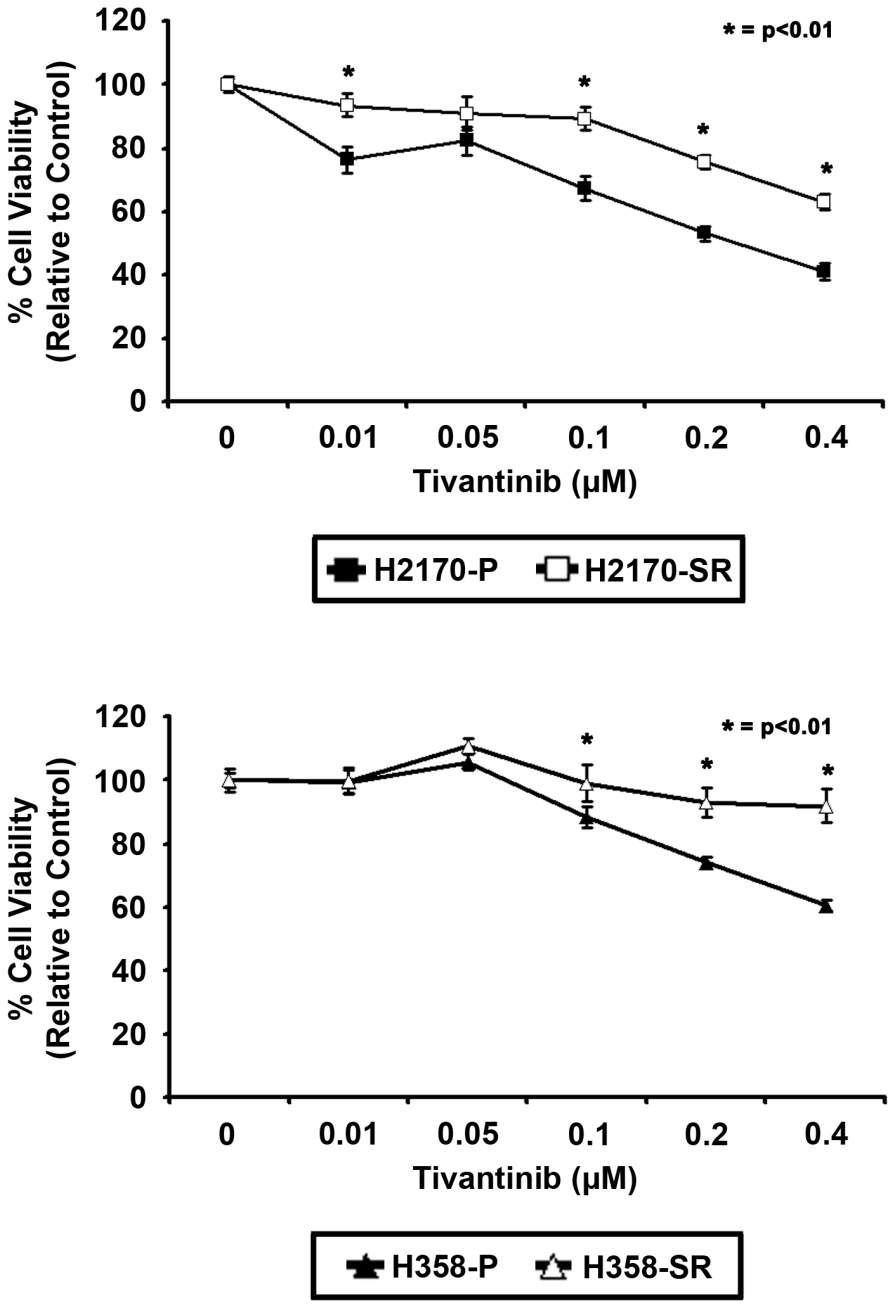
MTT assay showing differential response between parental and resistant NSCLC cell lines. H2170 and H358 cells were treated with tivantinib (0.01–0.4 µM) for 24 hours, tivantinib was removed, and cells were incubated for 72 hours, after which MTT viability assay was performed. SR H2170 cells showed a 3.2-fold decrease in sensitivity to the anti-proliferative effect of tivantinib at 0.1 µM tivantinib compared with parental cells. A 3.7-fold decrease in growth inhibition was also observed in SR H358 cells with 0.2 µM tivantinib compared to parental cells. Data shown are representative of three independent experiments showing similar results (n = 6, p<0.01).

The effect of tivantinib, an oral c-Met TKI currently in clinical trials [12], [14], on inhibition of cell growth in parental and resistant cells was investigated. Cells were treated with varying concentrations of tivantinib for 24 hours, after which the drug was removed [12]. Cells were then washed and incubated for an additional 72 hours and finally an MTT viability assay was performed. As shown in **Fig. 1**, at 0.1 µM tivantinib, H2170 parental cells were inhibited by 32% in comparison to untreated parental cells, while resistant cells were only inhibited by 10% in comparison to untreated resistant cells (p<0.01). Munshi et al have also shown sensitivity to submicromolar concentrations of tivantinib in NSCLC as observed in our studies [12]. A 3-fold decrease in inhibition was observed in H2170 resistant cells compared to parental cells (n = 6, p<0.01). In SR H358 cells treated with 0.2 µM tivantinib, a 3.7-fold decrease in inhibition was seen in resistant cells compared to parental cells (n = 6, p<0.01) (Fig1B). These data suggest that SR cells are also resistant to tivantinib.

### The T790M secondary mutation is not necessary for erlotinib resistance

Results of DNA Sanger sequencing of PCR products of exons 18–21 from H2170 and H358 parental and resistant cells showed no secondary erlotinib/gefitinib T790M or D761Y resistance point mutations [41]. These results confirm that our cells do not have known secondary mutations that would cause resistance. Thus, the mechanism by which they are resistant may be due to alternative signaling through receptors other than EGFR.

### Effect of EGF and erlotinib on EGFR phosphorylation and signaling proteins in two resistant NSCLC models

**Figure 2:**
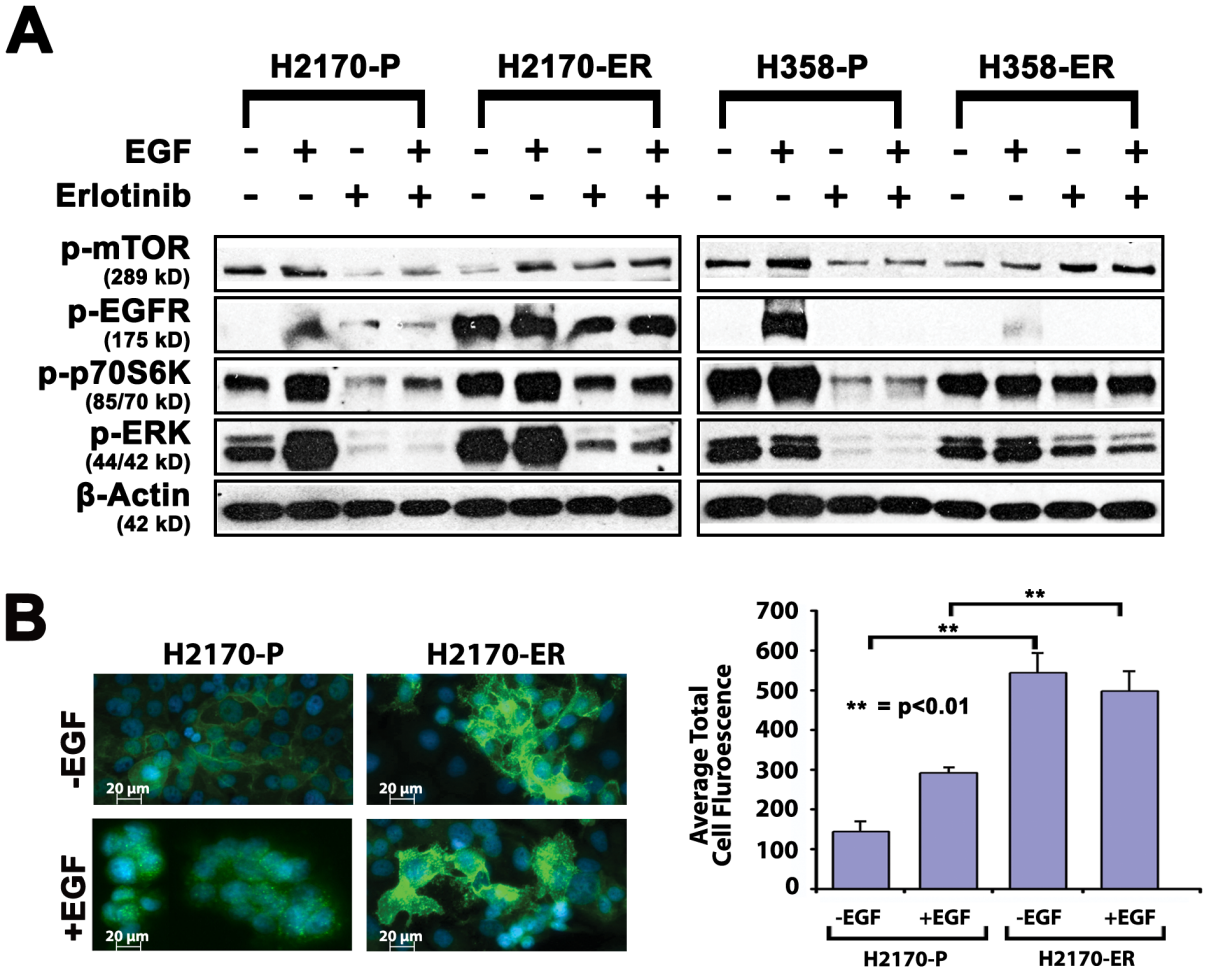
Differences in protein expression between parental and erlotinib resistant cell lines by western blotting. A. EGFR is autophosphorylated in ER H2170 and downregulated in H358-E4 resistant cell lines. p-mTOR (S2448) and its downstream signaling protein phospho-p70S6K (T389) are upregulated in both resistant cell lines. H2170 and H358 parental and resistant cell lines were starved overnight in 0.5% BSA and then treated with or without 7.0 µM of erlotinib for 24 hours and cells were stimulated with 10 ng/mL of EGF for 2.5 minutes. Higher concentrations of erlotinib were used since these NSCLC cell lines have no EGFR TK mutation. Autophosphorylation of EGFR on Y1068 was seen in the absence of EGF in ER H2170 cells which was not seen in ER H358-E4 cells. Upregulation of p-mTOR and its downstream protein phosho-p70S6K (T389) is seen in H2170 resistant lines +/− erlotinib. ER H2170 cells show increased EGFR phosphorylation +/− EGF. Upregulation of p-ERK (2–5-fold) was also seen in ER H2170 and H358 cells in +/− erlotinib B. To confirm autophosphorylation of EGFR, cells were plated on chamber slides, allowed to adhere for 24 hours and then starved overnight. Cells were then treated with +/− EGF for 15 minutes, fixed with acetone: methanol and visualized with p-EGFR (Y1068) primary antibody and anti rabbit DyLight secondary antibody (Thermo Fisher Scientific) (green) or Hoechst dye for nuclear staining (blue) on a Zeiss Axio Observer Z1 fluorescent microscope. Graph showing relative average total cell fluorescence units per 8 microscopic fields. There was a 3.8-fold increase in fluorescence when comparing parental to resistant cells in the absence of EGF in H2170 cells.

In order to understand the mechanism of erlotinib resistance, we compared two different erlotinib resistant cell lines, H2170 ER and H358 ER, with their respective parental cell lines. Cells were treated with either diluent, EGF, erlotinib or EGF+erlotinib. Erlotinib resistant (ER) H2170 cells appeared to exhibit constitutively autophosphorylated EGFR (Y1068) in the absence of its ligand, EGF (19-fold increase), while ER H358 cells exhibited a 6-fold decrease in p-EGFR (Y1068) in the presence of EGF (**Fig 2A**). These results were corroborated by immunofluorescence which demonstrated a minimal effect of EGF on EGFR phosphorylation in ER H2170 cells. After treatment of +/− EGF, H2170 parental and H2170-ER cells were stained using a specific primary anti-phospho EGFR (Y1068) antibody and DyLight 488-Conjugated Goat Anti-Mouse Secondary Antibody phosphorylated EGFR (green) and nuclei were stained blue with Hoechst dye. This suggests autophosphorylation of EGFR (**Fig 2B**). When total fluorescence units were measured, a 3.8- and 1.7-fold increase in fluorescence was observed in the presence and absence of EGF respectively in resistant cells as compared to parental cells (n = 8, p<0.01). Interestingly, there was no significant difference in fluorescence in H2170 ER cells in the presence and absence of EGF (p<0.01).

We further studied the effect of erlotinib resistance on the mTOR pathway, a key regulator of cancer cell growth [42], by measuring p-mTOR and its downstream substrate p-p70S6K. In ER H358 and H2170 cells, upregulation (2–4-fold) of p-mTOR was observed in the presence of erlotinib (**Fig 2A**). Additionally, upregulation (2-fold) of p-p70S6K was also observed in ER H2170 and H358 cells in the presence of erlotinib. Further, p-ERK was also upregulated (2–5-fold) in ER H2170 and H358 cells in the presence and absence of erlotinib (**Fig 2A**). No modulation of total mTOR, EGFR, p70S6K or ERK was observed (**Fig S1**). Our results indicate that the mTOR pathway and other receptors could upregulate p-p70S6K thereby mediating resistance through two separate mechanisms in H2170 and H358 NSCLC models.

### Effect of HGF and SU11274 on c-Met phosphorylation and signaling proteins in two NSCLC models

**Figure 3:**
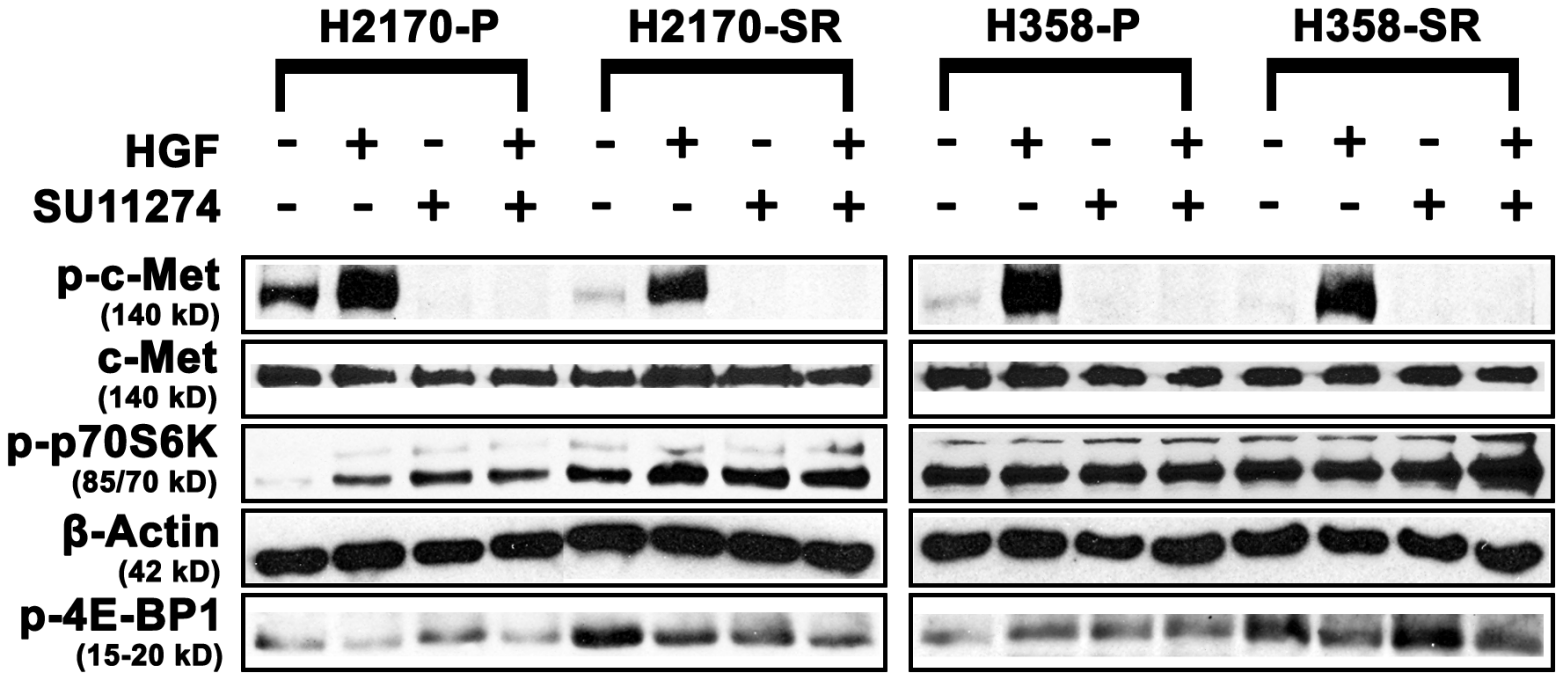
Differential expression of mTOR pathway proteins in parental and SU11274 resistant H2170 and H358 cell lines by western blotting. Cells were starved overnight and then treated with or without 8.0 µM SU11274 for 24 hours. Cells were stimulated with 40 ng/mL of HGF for 2.5 minutes after which western blot analysis was performed. Downregulation of p-c-Met (Y1003) was seen in both cell lines. Upregulation of p-p70S6kinase (S371) was observed in SR H2170 cells. Upregulation of p-4E-BP1 (T37/46) was also observed in both cells lines +/− SU11274.

To understand the resistance mechanism to c-Met inhibitors, we established SR H2170 and SR H358 cell lines and treated them with diluent, HGF, SU11274 and HGF+SU11274. SR H2170 and SR H358 cells exhibited a 4- and 1.5-fold downregulation of p-c-Met (Y1003) respectively, with no changes in total c-Met levels as analyzed by western blotting (**Fig 3**). Downregulation appears to be totally independent of any SU11274 treatment since the downregulation was observed after six passages in the absence of the drug. This could indicate that SR H2170 and H358 do not utilize p-c-Met as a means of resistance which would suggest a separate mechanism. Similar to ER cells, in untreated SR H2170 cells, we found a marked upregulation (20-fold) of p-p70S6K, a protein downstream of mTOR that is involved in cancer cell survival [42], and an upregulation was seen in cells treated with HGF and SU11274 (**Fig 3**). A 2-fold upregulation in p-4E-BP1, protein downstream of mTOR that promotes tumorigenicity, was observed in both SR H2170 and H358 cells (**Fig 3**). No modulation of total mTOR, EGFR, p70S6K or ERK was observed in either cell line (**Fig S1**). These results indicate that the mTOR pathway may be involved in mediating resistance.

### Activation of the Wnt pathway contributes to EGFR/c-Met TKI resistance

**Figure 4:**
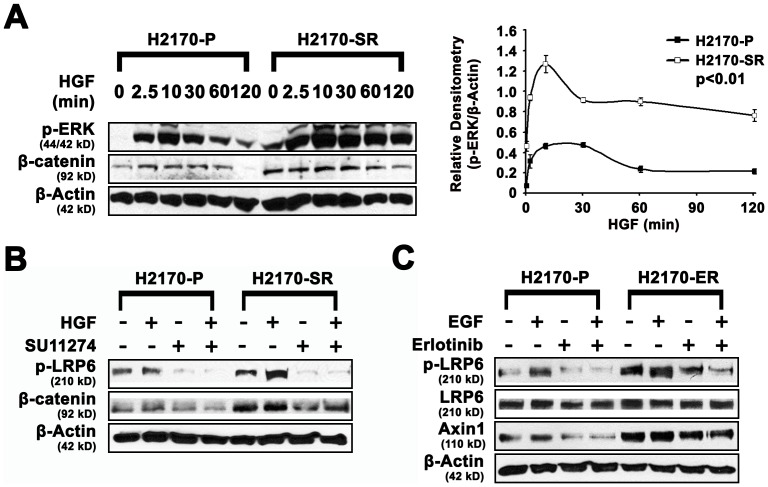
Differential expression of ERK/Wnt pathway proteins in parental and SU11274/Erlotinib resistant H2170 cells by western blotting. A. In SR H2170 cells, HGF induced pronounced p-ERK signaling compared to parental cells. Cells were starved for 48 hours and then stimulated with 40 ng/mL of HGF. Western blotting in SR H2170 indicated that, HGF activated p-ERK (T202/Y204) remained high for 120 minutes compared to parental lines. Basal levels of active β-catenin were also 2-fold higher and remained high (3.6-fold) for 120 minutes after HGF treatment in SR H2170 cells compared to those in parental cells over 60 minutes incubation. These experiments were done in triplicate. Relative densitometry of p-ERK/β-actin in SR H2170 cells was depicted which is an average of three independent experiments (n = 3, p<0.01). B. Regulation of proteins in the Wnt signaling pathway after treatment of H2170 with SU11274. Upregulation of pLRP6 (2 to 3.0-fold) and β-catenin (3 to 8.0-fold) were seen in resistant H2170 cells in the presence or absence of SU11274. C. Regulation of proteins in the Wnt signaling pathway after treatment of ER H2170 cells with erlotinib. Upregulation of LRP6 (2 to 5-fold), and Axin1 (2 to 3.5-fold) were seen in resistant H2170 cells in the presence or absence of erlotinib.

The Wnt pathway regulates cellular proliferation and plays a key role in development of lung cancer [43], [44]. Since β-catenin signaling was shown to activate the ERK signaling pathway [45], we examined p-ERK (T202/Y204) and active β-catenin in response to HGF over time in SR H2170 cells. We found that p-ERK remained elevated for greater than 120 minutes in SR H2170 cells but only for 30 minutes in parental cells (**Fig 4A**). Interestingly, in non-stimulated cells, basal levels of active β-catenin (2-fold) and p-ERK (5.6 fold) were higher and remained elevated for 120 minutes after HGF treatment in SR H2170 cells compared to parental cells after a 60 minute incubation (n = 3, p<0.01) (**Fig 4A**), which suggests crosstalk of the c-Met, mTOR and Wnt pathway. Following treatment with SU11274 and HGF, we observed a 3–8-fold increase in active β-catenin in the presence and absence of SU11274 in SR H2170 cells (**Fig 4B**). A 2–3-fold upregulation of p-LRP6 in the presence and absence of SU11274 was also seen. Upregulation of proteins associated with the Wnt pathway was confirmed in ER H2170 cells. We observed a 2–4-fold increase and 3–5-fold increase of p-LRP6 in the absence or presence of erlotinib, respectively. LRP6 phosphorylation may indicate activation of the Wnt pathway [46]. We also observed a 2–3.5-fold increase in expression of Axin1, a regulator of LRP6, and subsequently the Wnt pathway [31] (**Fig 4C**).

### The growth of H2170 and H358 combination resistant (CR) cells are inhibited by everolimus and XAV939

**Figure 5:**
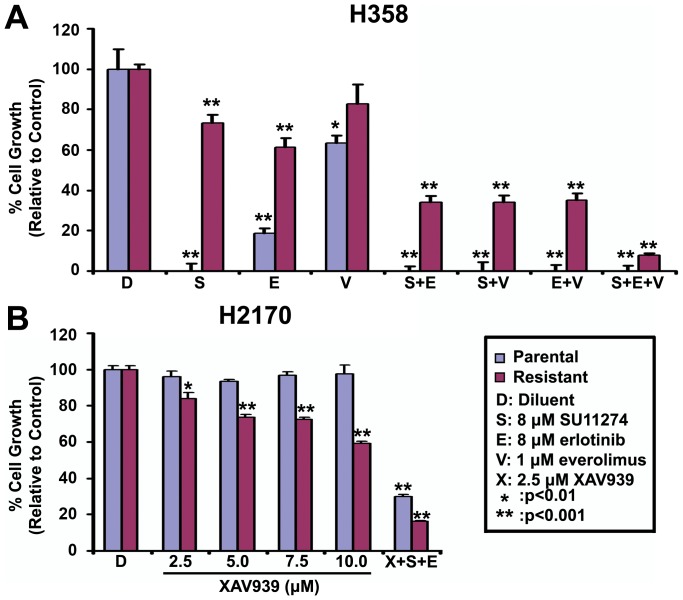
Growth of combination resistant (CR) cell lines is inhibited significantly by adding everolimus and XAV939 in the presence of SU11274 and erlotinib. Cells were treated for 96% growth inhibition was observed when everolimus was used with both SU11274 and erlotinib. B. Parental H2170 cells show little or no inhibition when given increasing concentrations of XAV939. Conversely, CR H2170 cells when treated with XAV939, were inhibited in a dose responsive manner. H2170 CR cells showing 40% inhibition to Wnt antagonist XAV939 (10 µM) alone, showed an 85% inhibition with triple combination of XAV939, SU11274 and erlotinib (p<0.01). Each experiment for each treatment condition was repeated three times.

Since the mTOR pathway is involved in anti-cancer drug resistance [47], sensitivity to mTOR inhibition in CR H2170 and H358 cell lines was tested. Treatment with 1 µM everolimus inhibited H358 parental cells by 40% and resistant cells by only 20%. Interestingly, the same concentration of everolimus inhibited the growth of parental cells completely and resistant cells by 95% when used in combination with either SU11274 (8 µM) or erlotinib (8 µM) (**Fig 5A**). Similar results were found in CR H2170 cells (99% inhibition of growth, data not shown). We then tested the effect of Wnt inhibition in resistant cells. H2170 parental and CR cell lines were treated with increasing concentrations of XAV939 and an MTT viability assay was performed. Interestingly, parental cells showed little or no response to XAV939, however, CR cells were inhibited in a dose responsive manner (**Fig 5B**). Furthermore, when XAV939 was combined with SU11274 (8 µM) and erlotinib (8 µM), an 85% decrease in viability was observed in CR cells. This suggests that Wnt signaling has a major role in resistant cells.

## Discussion

Molecularly targeted TKIs have become integral to the therapy used by clinicians to combat previously untreatable NSCLC. However, acquired resistance to TKIs has severely limited the extent to which this therapy can be employed effectively. Contrary to previous studies, which have focused on EGFR mutations [8], [48], we investigated possible alternative signaling pathways in drug resistant cell lines. We studied two NSCLC model cell lines which showed either upregulation (H2170) or downregulation (H358) of p-EGFR and downregulation p-c-Met (H2170 and H358). Resistant cells did not display either the T790M or D761Y mutations, suggesting the use of an alternative signaling mechanism to overcome erlotinib susceptibility. Interestingly both H2170 and H358 resistant cells display upregulation of the mTOR pathway. Moreover, H2170 resistant cell lines showed modulation of both the mTOR and Wnt pathways which suggests their roles in the mechanism of resistance.

Currently no link has been established between c-Met TKI resistance and mTOR in NSCLC. However, earlier studies suggest that inhibition of c-Met kinase activity leads to reduced activation of the PI3K/AKT/mTOR pathway in transformed cells [25]. However, in the present study, we observed no phosphorylation of c-Met in H2170 and H358 resistant cell lines when treated with SU11274. This suggests that SU11274, while an effective inhibitor of c-Met phosphorylation, has little effect on the inhibition of mTOR and its downstream signaling pathways necessary for cell growth and survival in resistant lines [25]. Since resistant cell lines have been shown to proliferate in the presence of SU11274, we suggest alternative pathways have a major role in overcoming c-Met inhibition and additional molecular targeting may be necessary to inhibit cell growth. The role of the mTOR pathway in resistance mechanisms is evidenced by a 2–4-fold increase of p-mTOR in resistant H2170 and H358 cells compared to parental cells in response to erlotinib treatment. Moreover, p-p70S6K, and p-4E-BP1 are also upregulated in resistant cell lines, thus the mTOR pathway appears to be strongly activated when exposed to EGFR/c-Met TKIs. Surprisingly, inhibition of mTOR alone did not significantly inhibit the growth of H358 and H2170 resistant cell lines. However, when used in combination with EGFR/c-Met TKIs, resistance was overcome, suggesting a link to the mTOR pathway, which is consistent with previous studies [49], [50]. Another study found synergistic effects with an EGFR and mTOR inhibitor combination in T790M positive NSCLC cells [51]. However, our results demonstrate a clear link between non-phosphorylated EGFR (T790M negative), c-Met inhibitor resistance and the mTOR pathway in NSCLC. This study indicates that targeting of the mTOR pathway could be an effective therapy in NSCLC patients, irrespective of EGFR secondary mutations. Additionally, upregulation of active β-catenin in SR H2170 cells was observed. Furthermore, we also observed upregulation of p-LRP6 and Axin1 in the presence and absence of erlotinib, which suggests activation of the Wnt pathway in ER H2170 cells [46]. Interestingly, sensitivity to Wnt inhibition appears to be selective to resistant lines, since we show that XAV939 had minimal effect on parental cell viability but significantly inhibited resistant cells at the same concentrations. This result implicates Wnt as an essential pathway in EGFR/c-Met TKI resistance. Conversely, everolimus showed greater efficacy on resistant lines when used in combination with erlotinb and SU11274. Thus, our results may apply to patients with acquired resistance to a combination of erlotinib and tivantinib [52], as our cells demonstrate decreased sensitivity to tivantinib.

Previous studies have shown that an EGFR T790M mutation is the primary cause of resistance [8] and many have focused on developing irreversible TKIs against EGFR that would prevent or maintain efficacy against the T790M mutation [53]. Unfortunately, to date, no therapy has been approved which can effectively counteract the T790M mutation [53]. Conversely, this study focused on alternative signaling pathways that appear to be involved in EGFR/c-Met resistant cell lines. By targeting alternative signaling pathways that are downstream or parallel to EGFR, we have shown decreased cell viability of TKI resistant cells. When translated into clinical practice, this novel therapeutic approach may have the potential to increase patient PFS. Targeting additional pathways other than EGFR in NSCLC patients has already been shown to be effective in clinical trials combining erlotinib with tivantinib or MetMab [14], [15]. Therefore, concurrent targeting of mTOR and Wnt pathways may further improve drug efficacy and prevent resistance. Furthermore, in the event that the T790M mutation can be targeted by TKIs, additional pathways such as mTOR and Wnt may cause additional tumorigenicity even after downregulation of p-EGFR.

In previous studies, Wnt signaling has been shown to be involved in NSCLC development, and when hyperactive, it can modulate the mTOR pathway and play a role in tumorigenicity [54], [55]. Since EGFR and Wnt signaling are known to exhibit crosstalk, activation of the Wnt pathway may stimulate activation of EGFR [31], [32]. Moreover, Wnt is able to transactivate EGFR and the MAPK pathway through the Wnt/Fz/LRP pathway [56]. This could be a possible explanation of constitutive phosphorylation of EGFR in H2170 resistant cells, despite the absence of any secondary resistance inducing mutation. Although Axin1 is normally defined as a negative regulator of the Wnt pathway, recent studies indicate that sequestration of Axin1 with LRP6 stimulates the Wnt pathway [57], [58]. Similar to the results with mTOR inhibition, Wnt inhibition using XAV939 resulted in a significant reduction in viability of H2170 resistant cells, suggesting that targeting the Wnt pathway may be a viable option for treating EGFR/c-Met TKI resistance in NSCLC. In resistant H358 cell lines, the mTOR pathway could be activated through ERK [59], and therefore use a different resistance mechanism.

While currently no link between Wnt and c-Met is established in NSCLC, a connection between c-Met and non-canonical Wnt/β-catenin has been shown in melanoma [60]. However, the mechanism whereby the Wnt pathway contributes to EGFR/c-Met TKI resistance is currently unknown. It was shown that Cav-1 interacts with LRP6 resulting in stimulation of Akt/mTORC1 signaling in prostate cancer [61]. We speculate that the Wnt pathway, which is known to activate mTOR [54], may be working synergistically with the mTOR pathway by using proteins common to both, such as LRP6 to increase tumorigenicity in H2170 resistant cells. The constitutive phosphorylation of p-EGFR could activate Ras/Raf/MAPK pathway, resulting in upregulation of p-ERK that may phosphorylate GATA-6, which may in turn stimulate transcription of Wnt7b, a known canonical Wnt pathway activator [62]–[64]. These studies suggest a novel mechanism of resistance in NSCLC cells which is currently being further investigated.

Our studies provide evidence that the mTOR and Wnt signaling pathways contribute to acquired EGFR/c-Met TKI resistance. Additionally, combination therapy may be a potential way to prevent secondary resistance in many lung cancer patients [50]. Inhibition of either mTOR or Wnt signaling pathways in NSCLC sensitizes cells to EGFR/c-Met TKIs, thus restoring their efficacy. Studies involving *in vivo* experiments comparing parental and resistant cells will be needed to confirm our current findings. Developing new therapeutics that target multiple RTKs might be another approach in addition to the presently used inhibitors [49], [50].

In summary, our studies suggest that EGFR/c-Met TKI mechanisms of resistance act through the Wnt and mTOR signaling pathways. In NSCLC Wnt and mTOR may contribute to EGFR and c-Met signaling, as in the case of H2170 resistant cells, or mTOR could replace EGFR and c-Met signaling as in the case of H358 cells, allowing for enhanced survival and proliferation. To our knowledge, this is the first study showing a relationship between the mTOR and Wnt signaling pathways and acquired EGFR/c-Met TKI resistance. We suggest a novel treatment modality to overcome the acquired resistance seen in NSCLC. Additional studies on GATA-6/Wnt and mTOR signaling pathways are currently in progress and crosstalk between EGFR and c-Met and simultaneous treatment with their ligands and inhibitors are also being investigated.

## Supporting Information

Figure S1 **Expression of unphosphorylated total proteins in erlotinib resistant (ER) H2170 and H358 cells in the presence and absence of erlotinib and EGF.** No change was observed in the expression of total mTOR, EGFR, ERK, p70S6Kinase, β-actin with or without EGF and/or erlotinib.
(TIF)
