# Supplementary figures and images for: Alternative Signaling Pathways as Potential Therapeutic Targets for Overcoming EGFR and c-Met Inhibitor Resistance in Non-Small Cell Lung Cancer
Source: PLoS One. 2013 Nov 4;8(11):e78398. doi: 10.1371/journal.pone.0078398 (PMC3817236; doi:10.1371/journal.pone.0078398)

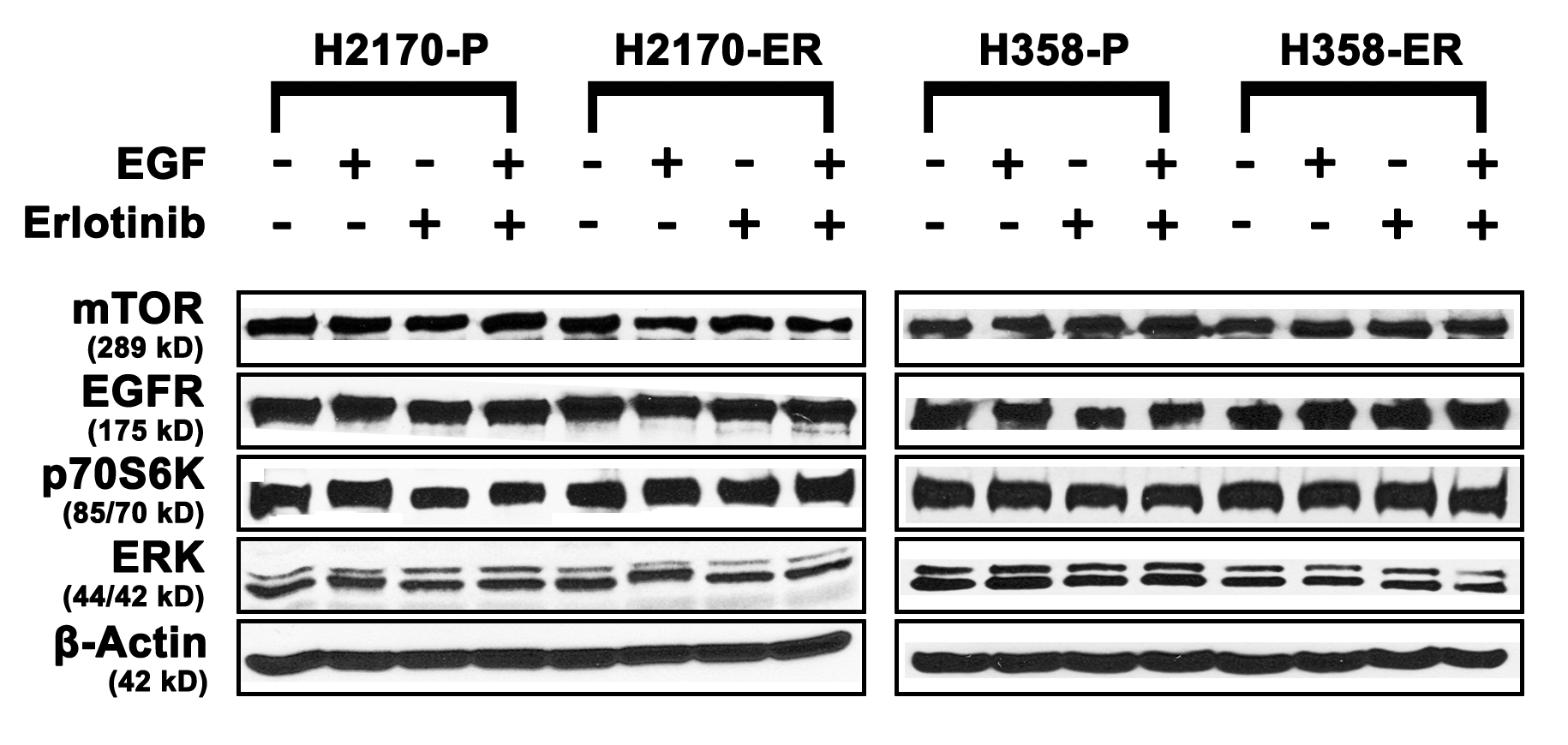

Supplement: Figure S1 — Expression of unphosphorylated total proteins in erlotinib resistant (ER) H2170 and H358 cells in the presence and absence of erlotinib and EGF. No change was observed in the expression of total mTOR, EGFR, ERK, p70S6Kinase, β-actin with or without EGF and/or erlotinib. (TIF) [file pone.0078398.s001.tif]
